# Effect of voice training in the voice rehabilitation of patients with vocal cord polyps after surgery

**DOI:** 10.3892/etm.2014.1499

**Published:** 2014-01-23

**Authors:** LI LIN, NA SUN, QIUHUA YANG, YA ZHANG, JI SHEN, LIXIN SHI, QIN FANG, GUANGBIN SUN

**Affiliations:** 1 Department of Otolaryngology, Gongli Hospital, Shanghai 200135, P.R. China; 2 Department of Nurse Care, Gongli Hospital, Shanghai 200135, P.R. China

**Keywords:** polyp of vocal cord, laser, surgery, voice training, voice evaluation

## Abstract

The objective of the present study was to determine the effect of voice training on the vocal rehabilitation of patients with vocal cords polyps following phonomicrosurgery. A total of 60 cases of vocal cord polyps treated by laser phonomicrosurgery were randomly divided into training and control groups with 30 cases in each group. The patients were treated with laser phonomicrosurgery, routine postoperative treatment and nursing. The training group were additionally treated with vocal training, including relaxation training, breathing training, basic pronunciation training, chewing voice training and tone sandhi pronunciation training, and attention was paid to the training steps. Subjective and objective voice evaluations of the two groups were compared three months after the surgery and the differences between groups were statistically significant (P<0.05). Voice training may significantly improve the postoperative voice quality of patients with vocal cord polyps and support rehabilitation.

## Introduction

Voice disorder is a general term for hoarseness as the main symptom of laryngeal lesions; it seriously affects the life quality of patients and interpersonal communication. At present, treatment is mainly comprehensive therapy, including conservative internal medical treatment and surgical interventions. Conservative treatments are mainly based on voice rest, drug therapy and voice therapy (i.e., voice training) (1). At present, due to its low cost, reduced pain, rapid recovery, preservation of laryngeal function and other advantages, laser phonomicrosurgery is increasingly recognized as an ideal method for the treatment of benign and malignant vocal cord lesions (2). However, after surgery certain patients continue to have functional phonation disorders, the key cause of which is the improper use of the voice (2). Vocal rehabilitation following surgery includes two processes, which are healing of the wound caused by surgery and vocal function recovery. Voice training enables patients to establish or re-establish the physiological balance among the vocal organs and to correct the erroneous conditioned reflex caused by the vocal cord lesions. The present study applied vocal training in patients with vocal cord polyps following laser phonomicrosurgery, compared subjective and objective voice evaluation parameters before and after treatment, and investigated the therapeutic effect of vocal training.

## Subjects and methods

### General data

Between January 2011 and December 2012, 60 patients with vocal cord polyps from the Department of Otolaryngology of Gongli Hospital (Shanghai, China) were enrolled for treatment by a group of doctors for a period of more than half a year. The patients presented with unilateral vocal cord polyps and underwent phonomicrosurgical CO_2_ laser surgery (40C, single pulse mode, 2 W; Lumenis Ltd., Yokneam, Israel). There were 33 male and 27 female cases, with an average age of 44.5 years (range, 30–68 years). Patients with bilateral vocal cord polyps, vocal leukoplakia, laryngeal cancer with respiratory or cardiovascular system diseases, and cardiovascular disease were excluded.

The patients were randomly divided into the training and control groups with 30 cases in each group. Each group had the same cultural background, severity and course of disease. This study was conducted in accordance with the Declaration of Helsinki and with approval from the Ethics Committee of Gongli Hospital. Written informed consent was obtained from all participants.

### Training methods

The two groups of patients were treated with routine laser phonomicrosurgery postoperative treatment and nursing, including postoperative silence for 1 week, voice rest for 1 month (including relative voice rest, talking as little as possible and speaking gently) (3), avoidance of spicy, dry and irritating food, no consumption of alcohol, tobacco, hot or cold water and acceptance of inhalation treatment using 80,000 U Gentamicin combined with 5 mg dexamethasone, twice a day, for 5 days. In addition, the training group received additional voice training, as follows (4): i) Education prior to training: Patients were made aware of the principle of vocalization and the necessity of voice training, to relieve postoperative anxiety and encourage active cooperation with this study. ii) Relaxation training: Patients adopted a supine position with their arms at the sides of the body, consciously gradually relaxed from the head to the trunk and limbs, and established a regular and slow respiratory rhythm through abdominal breathing. iii) Breathing training: Patients relaxed the body while standing with feet apart, inhaling deeply through the nose and exhaling with the lips slightly open. The abdominal breathing was taken slowly and evenly. iv) Basic pronunciation training: Natural yawning and sighing was performed. Patients sensed the mouth, pharynx and larynx opening and the larynx lowering during inhalation and the oral cavity, laryngeal muscles and jaw relaxing following inhalation. The vocal cords were blown open with a weak, uniform continuous airflow through the nose, the mouth was opened steadily as if blowing a bubble, and pronouncing [a:], [i:] and [u:] 3 single voices. Following a 30-sec rest, the process was repeated. v) Chewing voice training: The mouth was closed and chewing was performed with upward and downward movements of the tongue and jaw. The patients then opened the mouth wide, enabling the mouth, tongue and jaw to make more substantial movements, such as chewing and pronouncing [a:], [i:] and [u:], the 3 single voices. vi) Tone sandhi pronunciation training: After basic pronunciation and chewing voice training, the patients practiced tone sandhi and pronouncing [a:], [i:] and [u:], the 3 single voices, repeating from low to high, then from high to low in volume.

### Training steps

From the second day after the surgery, the patients started relaxation training for 15 min/time, 3 times a day and breathing training for 15 min/time, 3 times a day for 5 days. From the sixth day, after relaxation training and breathing training for 2 min each, the patients started to add basic pronunciation for 2 min, 2 cycles per time, 3 times per day. At the eighth to fourteenth day following surgery, the number of cycles was increased by one per day each day. From the fifteenth day after surgery, after the relaxation training, breathing training and basic pronunciation training, the patients added chewing voice training and the tone sandhi pronunciation training for 3 cycles, 3 times a day until 4 weeks after surgery. The patients visited the Gongli Hospital to perform the vocal training once each week, and practiced by themselves the rest of the time. Telephone follow-ups were conducted until 4 weeks after discharge, once or twice each week. The patients were provided with understanding, supervision and guidance relating to the vocal training and voice health, and were reminded of hospital visit times.

### Preoperative and postoperative voice evaluation

All patients accepted the subjective and objective evaluation 2 days before surgery and at 1 and 3 months after surgery. Subjective assessment: the patient self-assessment Voice Handicap Index (VHI) (5) is a measure of the functional, physical and emotional state of the patient, and provides an overall evaluation of the effect of an abnormal voice on the quality of life. Each state was divided into 10 problems, each of which was scored by the patient as follows: 0 = never, 1 = rarely, 2 = sometimes, 3 = often and 4 = always. The total score was 0–120 points; the higher the score the more serious the patient’s subjective assessment of their dysphonia. The patient voices were also assessed using the GRBAS scale. This comprises grading criteria for hoarseness assessment proposed by the Japanese Society for Logopedics and Speech Phoniatrics (6) and evaluates: A total hoarseness grade (G), the classification of overall perception of abnormal voice; the roughness (R), degree of irregular pronunciation; the breathiness (B), degree of breath sound; asthenia (A), weak or non-existent pronunciation; strain (S), excessive tension or forced pronunciation. Each evaluation of the five parameters was divided into 4 grades: grade 0, normal; grade 1, mildly abnormal; grade 2, moderately abnormal; grade 3, severe abnormalities. Patients were inquired with medical history with the same content and method, answering with the most natural tone and volume, in an examination room with an environmental noise level of <45 dB (SPL). Voices were scored by three professional phoniatricians working in voice therapy and the grades were averaged. Objective evaluation: The Computerized Speech Lab (CSL) model 4150 system (Kay Elemetrics Corporation, Montvale, NJ, USA) multi-dimensional voice program (MDVP in Kay Computer Speech Lab software) was used to conduct voice spectrum analysis. Evaluated parameters included fundamental frequency perturbation (jitter), amplitude perturbation quotient (shimmer), pitch perturbation quotient (PPQ), amplitude perturbation quotient (APQ), noise and harmonic ratio (NHR), and maximum phonation time (MPT).

### Statistical analysis

Using a Student’s t-test, data were analyzed using SPSS 11. 5 software (SPSS, Inc., Chicago, IL, USA). P<0.05 was considered to indicate a statistically significant result.

## Results

The subjective and objective test data of the two groups of patients before and after surgery are shown in Tables I and II. The subjective and objective voice evaluation indices of the patients showed that one month after surgery, certain indices of patients in the training group were superior compared with those of patients in the control group (P<0.01). Three months after surgery, all the indices of the training group were superior compared with those of the control group, and the differences were statistically significant (P<0.01). It was demonstrated, from the subjective and objective data of the two groups before and after surgery in this study, that the vocal rehabilitation effect following surgery in the patients of the training group was better than that of the control group, and the difference was statistically significant (P<0.05).

## Discussion

Following treatment by phonomicrosurgery, voice disorders require long-term voice modification treatment. Europe, the United States of America and Singapore have speech pathologists who are mainly devoted to the diagnosis, research and treatment of communication obstacles. However, until now in China the development of voice training remains relatively weak. The associated voice therapy is biased toward surgical treatment and treatment in the hospital with a lack of attention to voice nursing, and maintenance following surgery is not comprehensive or appropriate (7–10). The routine nursing care remains limited to diet guidance and voice rest.

The aims of voice training are to establish and re-establish the physical equilibrium among the relative vocal organs of the patients, and to modify the erroneous conditioned reflex caused by the vocal cord lesion. Through changing the vocal environment and condition of the patients, the voice training treatment helps to remove the control of the erroneous conditioned reflex, change vocal habits and establish a new conditioned reflex to enable restoration of normal phonation function (11,12). Relaxation training may enable the patient to be aware of the key point of tension, and to harmonize the association between the vocal organs and muscles throughout the whole body. Breathing training may make the breath even and smooth through counteraction between the two muscle groups of inhalation and exhalation. When the patient yawns, the Adam’s apple drops and the laryngeal muscle is relaxed. Therefore, the airway is fully open and the pharyngeal constrictor is relaxed. A sigh is formed by deep breathing. It may correct the high respiration and respiratory muscle tension, and achieve the best pronunciation state of the larynx. Basic pronunciation training coordinates with breathing, which may aid the dual band vibration and balance, strengthen the tension of the laryngeal adductor and thyroarytenoid muscles, and reduce the change in microvascular permeability. Chewing voice training may effectively alleviate the tension of the pronunciation and articulation muscles, enhance the pharyngeal resonance effect, and achieve a sound and air balance through the physiological function of natural chewing (13–17). In the present study, after voice training, the curative effect in the training group was consolidated, and recent and long-term vocal rehabilitation outcomes were superior to those of the control group. These results are consistent with the study by Cho *et al* (18).

This study applied vocal training in voice disorder patients following laser microsurgery to explore a new method of vocal training in clinical application. The GRBAS and VHI subjective assessments and the multiple objective indices, including jitter, shimmer, fundamental frequency perturbation quotient, APQ, NHR and MPT (19–26), were applied to comprehensively evaluate voice quality before and after surgery, and to conduct a comprehensive and scientific evaluation of the value of vocal training in the voice rehabilitation of vocal disorder following laser treatment. The present study measured the subjective and objective data of the two groups prior to and following surgery and showed that the vocal rehabilitation effect after surgery in the training group was better than that of the control group.

Vocal training application in the voice rehabilitation of patients with voice disorders following laser phonomicrosurgery is in compliance with the principles of the treatment of voice disorders, and may help patients with postoperative voice rehabilitation. The effects of the method are simple and satisfactory, and may reduce the economic burden and physical pain of postoperative voice rehabilitation.

## Figures and Tables

**Table I:** Comparison of preoperative and postoperative subjective evaluation indices of the two groups (mean ± SD).

	GRBAS scale			
		
Evaluation time group	G	R	B	VHI
Preoperative				
Control group	2.31±0.55	2.52±0.43	3.22±0.82	32.81±17.11
Training group	2.20±0.72	2.41±1.22	3.11±0.93	31.51±12.23
t-value	0.67	0.47	0.49	0.43
P-value	>0.05	>0.05	>0.05	>0.05
One month after surgery				
Control group	2.22±0.41	2.42±0.43	2.73±0.44	30.32±8.83
Training group	2.03±0.64	2.13±1.02	1.87±0.58	19.32±6.22
t-value	1.37	1.44	6.47	5.58
P-value	>0.05	>0.05	<0.01	<0.01
Three months after surgery				
Control group	2.03±0.42	2.33±0.41	2.44±0.52	27.21±4.13
Training group	1.02±0.41	1.13±0.52	0.61±0.23	9.41±6.03
t-value	9.18	9.92	17.60	13.34
P-value	<0.01	<0.01	<0.01	<0.01

GRBAS scale, grading criteria for hoarseness assessment presented by Japanese Society for Logopedics and Speech Phoniatrics; G, total hoarseness grade; R, roughness; B, breathiness; VHI, Voice Handicap Index.

**Table II:** Comparison of preoperative and postoperative objective evaluation indices of the two groups (mean ± SD).

Evaluation time group	Jitter (%)	PPQ (%)	Shimmer (%)	APQ (%)	NHR	MPT (sec)
Preoperative						
Control group	4.50±0.93	3.36±1.05	6.84±1.24	6.76±0.88	0.51±0.05	9.92±2.25
Training group	4.62±1.20	3.28±0.78	7.46±1.27	7.09±1.32	0.43±0.01	9.63±1.84
t-value	−0.43	−0.33	−1.91	−1.14	0.57	0.55
P-value	>0.05	>0.05	>0.05	>0.05	>0.05	>0.05
One month after surgery						
Control group	4.21±0.91	3.09±0.82	6.43±2.25	6.53±0.76	0.47±0.12	9.62±1.83
Training group	3.35±1.01	3.09±0.71	5.12±1.25	6.27±0.58	0.33±0.19	13.94±2.32
t-value	3.47	0.00	2.79	1.49	3.41	−8.01
P-value	<0.01	>0.05	<0.01	>0.05	<0.01	<0.01
Three months after surgery						
Control group	4.21±0.94	3.13±0.92	6.29±1.01	6.03±0.76	0.46±0.11	13.64±3.62
Training group	1.03±0.52	0.58±0.18	3.23±0.94	2.59±0.55	0.14±0.06	16.34±1.92
t-value	16.22	14.91	12.14	20.12	13.91	−3.61
P-value	<0.01	<0.01	<0.01	<0.01	<0.01	<0.01

Jitter, fundamental frequency perturbation; Shimmer, amplitude perturbation quotient; PPQ, pitch perturbation quotient;APQ, amplitude perturbation quotient; NHR, noise and harmonic ratio; MPT, maximum phonation time.
